# Formulating treatment of major psychiatric disorders: algorithm targets the dominantly affected brain cell-types

**DOI:** 10.1007/s44192-022-00029-8

**Published:** 2023-01-05

**Authors:** Jeffrey Fessel

**Affiliations:** Department of Medicine, University of California, 2069 Filbert Street, San Francisco, CA 94123 USA

**Keywords:** Pharmacotherapy, Psychiatric disorders, Brain cell-types

## Abstract

**Background:**

Pharmacotherapy for most psychiatric conditions was developed from serendipitous observations of benefit from drugs prescribed for different reasons. An algorithmic approach to formulating pharmacotherapy is proposed, based upon which combination of changed activities by brain cell-types is dominant for any particular condition, because those cell-types contain and surrogate for genetic, metabolic and environmental information, that has affected their function. The algorithm performs because functions of some or all the affected cell-types benefit from several available drugs: clemastine, dantrolene, erythropoietin, fingolimod, fluoxetine, lithium, memantine, minocycline, pioglitazone, piracetam, and riluzole

**Procedures/findings:**

Bipolar disorder, major depressive disorder, schizophrenia, Alzheimer’s disease, and post-traumatic stress disorder, illustrate the algorithm; for them, literature reviews show that no single combination of altered cell-types accounts for all cases; but they identify, for each condition, which combination occurs most frequently, i.e., dominates, as compared with other possible combinations. Knowing the dominant combination of altered cell-types in a particular condition, permits formulation of therapy with combinations of drugs taken from the above list. The percentage of patients who might benefit from that therapy, depends upon the frequency with which the dominant combination occurs in patients with that particular condition.

**Conclusions:**

Knowing the dominant combination of changed cell types in psychiatric conditions, permits an algorithmically formulated, rationally-based treatment. Different studies of the same condition often produce discrepant results; all might be correct, because identical clinical phenotypes result from different combinations of impaired cell-types, thus producing different results. Clinical trials would validate both the proposed concept and choice of drugs.

## Introduction

It is an old debate as to whether or not psychiatry is a branch of medicine or should be regarded as totally separate; also debated are queries concerning confidence in both diagnostic systems and treatment strategies [1, 2]. This article is not intended as a polemic in those debates but is aimed at a rational formulation of pharmacotherapy for patients whose cases receive only limited benefit from psychotherapy. It is relevant to note that effective pharmacotherapy for major psychiatric disorders commenced after serendipitous observations that chlorpromazine, originally produced and prescribed as an antihistamine, was seen to reduce symptoms of mania and schizophrenia; that imipramine, originally produced and prescribed as an antipsychotic, reduced depression; and that lithium reduced the excitability of guinea-pigs, leading to treatment of mania. Most subsequently developed antipsychotic drugs were based on those original observations, although a different approach was based upon the serotonin hypothesis and led to development of selective serotonin re-uptake inhibitors (SSRI). The serotonin hypothesis is now considered probably incorrect [3]; SSRI promotion of oligodendrocytes [4] and M2 microglia [5] is a possible basis for their efficacy in treating depression. During the past few decades, many research efforts have undertaken genetic studies of psychiatric disorders, in the expectation that their results might lead to rational drug development. Sullivan and Geschwind, however, stated that even with complete knowledge of the genetic architecture of a psychiatric disorder, full understanding requires knowing how loci interact in the nucleus, how gene and isoform expression are coordinated for many involved genes, and how all of these affect neural networks [6]. Likewise, in his editorial concerning genetic testing in psychiatry, Dubovsky pointed out that no genetic marker has yet been shown to be useful in prospectively identifying any specific psychiatric disorder [7] Further, virtually every major psychiatric condition has a pathogenesis that involves genetic and metabolic impairments plus environmental factors; addressing them all requires an impractical number of medications. That task may be greatly simplified by the algorithmic approach to formulating treatment that is proposed here, because (1) there are only five major families of brain cell-types, i.e., astrocytes, oligodendrocytes, neurons, endothelial cells, and microglia, and all of them contain the genetic, metabolic and environmental factors that have affected their function; and (2) eleven available drugs, described below, benefit the cell-types that are dominantly affected in those psychiatric disorders that are discussed here to illustrate the approach.

## The five families of cell-types in the brain

The following is a brief overview of the five families of cell-types in the brain. For several of them it is known that there are subtypes; but at this time there is little or no information about how pharmacotherapy affects those subtypes or their association with clinical outcomes. Acknowledging that the situation may change in the future when such data regarding subtypes becomes available, the present article, with a few exceptions, confines itself to the main families of cell-types and ignores the issue of subtypes. The cell-types are listed here uniformly, in the following order: astrocytes. Oligodendrocytes, neurons, endothelial cells, and microglia. Oligodendrocytes are always mentioned before neurons because if their numbers or functions diminish, then neurons and neural tracts suffer. Regarding endothelial cells: cerebral blood flow (CBF) is sometimes used as surrogate. For astrocytes. Oligodendrocytes, neurons, and endothelial cells, only down-regulation of their number or function is considered as pathogenic, since up-regulation is unlikely to have deleterious consequences; regarding microglia, it is up-regulation of number or function that is considered as most likely to have pathogenic consequences.

*Astrocytes* The high importance of astrocytes is shown by their accounting for approximately half the volume of the adult mammalian brain; that they provide the primary structural and trophic support for neurons; and that astrocyte processes ensheath neurons and capillaries. One astrocyte supports the functions of 3 or 4 neurons, and since each cortical neuron has approximately 38,000 synapses, processes from a single astrocyte can envelop approximately 150,000 synapses [8, 9]. The end feet of astrocyte processes, which contain aquaporin 4, also ensheath cerebral capillaries; aquaporin 4 must regulate that ensheathment, because aquaporin 4 knockout mice had ~ 60% less coverage of cerebral capillaries by astrocytic processes; for that reason, astrocytes regulate water permeability and, therefore, the transport of drugs across the blood–brain barrier (BBB) [10]. Other important functions of astrocytes are their uptake of the extracellular potassium from neural activity; their regulation of Ca^2+^ signaling and excitatory neurotransmitters; and their delivery of energy to neurons via the astrocyte-neuron lactate shuttle.

*Oligodendrocytes* A pool of undifferentiated oligodendrocyte precursor cells (OPC) remains in the adult CNS; they can differentiate into pre-myelinating and myelinating oligodendrocytes [11]. Myelination of axon is a major function of oligodendrocytes is myelination; its impairment would cause synaptic dysfunction, abnormal neural tracts, and disturbed cognition [12]. Phenotypic markers identify the lineage: OPC express platelet derived growth factor (PDGF); myelinating, mature oligodendrocytes express myelin basic protein (MBP) and myelin oligodendrocyte glycoprotein (MOG); and both OPCs and mature oligodendrocytes share markers such as OLIG2. Mature oligodendrocytes express all three types of glutamate receptors—AMPA, kainate, and NMDA, making them subject to excitotoxic death. Stressors affect oligodendrocyte maturation, which impacts neurons. The prefrontal cortex (PFC) of mice housed in isolation had oligodendrocytes with shorter processes and less branching; they had fewer myelin genes (*MBP* and *MAG*); and electron microscopy showed reduced myelin thickness [13]. Liu et al. subjected mice to three different stressors, and showed reduction of both myelin and oligodendrocyte gene transcripts [14]. Mice subjected to other mice that were aggressive, had significantly down-regulated genes that were myelin-related [15]; Banasr et al. subjected mice to a dozen different stressors, which resulted in a 39% reduction of oligodendrocytes [16].

*Neurons* need little elaboration since, obviously, their impairments are fundamental to the pathogenesis of almost every psychiatric condition. Already mentioned, a single astrocyte contacts thousands of synapses, and also the cerebral microvasculature [17]. In that way, there is a tricellular collaboration that incorporates neurons, astrocytes, and endothelial cells.

*Endothelial cells* The importance of endothelial cells is illustrated in the brain of AD patients, where string vessels, the remnants of capillary injury, are increased and vascular density is reduced (reviewed in ref [18]).

*Microglia* In the CNS, activated microglia may have a pro-inflammatory M1 phenotype or an anti-inflammatory M2 phenotype. M1 microglia produce cytokines and chemokines (IL-1β, IL-6, IL-12, TNF-α, CCL2), express NADPH oxidase, and generate reactive oxygen species (ROS) and reactive nitrogen species; M2 microglia produce anti-inflammatory cytokines (IL-10, TGF-β), growth factors (IGF-1, FGF, CSF1), and neurotrophic growth factors [19]. A skewed M1 activation over M2 has been related to disease progression in AD [20], and is often involved in other psychiatric conditions.

## Altered functional activity of dominant cell-types in selective psychiatric disorders used as illustrations

Although the concept suggested in this article is an algorithmic approach for formulation of pharmacotherapy of any psychiatric condition, five of those are considered for illustrative purposes. Decreases in either their number or activity, affecting astrocytes, oligodendrocytes, neurons, and endothelial cells, would predispose to disease, whereas for microglia, increase in their number or functional activity would predispose to disease; changes in opposite direction would likely not be deleterious and, therefore, are not mentioned here. Note that the determination of the dominant change in the brain cell-type is based upon the findings in citations provided by PubMed and Google Scholar, supplemented by those in key articles.

### Bipolar disorder (BD)

*Astrocytes* Liu et al. [21] found increases in 3 reports, and decreases in 1.

*Oligodendrocytes* Liu et al. found no increases in any report and decreases in 2 [21], and Uranova et al. saw a 29% decrease [22].

*Neurons* Gigase et al. summarized autopsies showing increased neurons in six and decreased neurons in nine [23]. In the dorsolateral (dl) prefrontal cortex (PFC) of BD, Rajkowska et al. saw reduced neuronal density in layers III and V (16% and 30%, respectively) [24]. Summarizing 12 reports, Cotter et al. found smaller neuronal size in layers 5 and 6 of the dlPFC [25]. Other studies of the dlPFC in BD, showed increased neurons in 1[26]and decreases in 12 [27–38].

*Interneurons* Gigase et al. summarized reported studies of BD brains, showing interneurons as increased in one brain region, and decreased in sixteen [23].

*Endothelial cells* Kruger et al. measured CBF by using [150] H_2_O and PET scanning [39]. Subjects drafted a brief script describing a sad life event; the script was projected onto a computer screen during the PET scan; CBF was measured in baseline euthymia and in provoked sadness. Decreased CBF in the ventromedial (vm) PFC distinguished BD patients from their at-risk siblings. Another study used breath-holding, which should produce decreased cerebral oxygenation and cerebral vasodilatation. Twenty-five adolescents with BD and 25 psychiatrically healthy controls completed six 15-s breath-holds during functional magnetic resonance imaging, and cerebrovascular reactivity (CVR) was determined by comparing blood-oxygenation-level dependent signal changes [40]. During breath-holding, CVR in several brain regions was decreased in BD versus controls.

A systematic review by Toma et al., yielded 33 studies, 118 subjects with BD/depression, 46 with BD/mania, 24 with BD/euthymia, and 385 controls [41]. CBF in multiple brain areas was reduced in all BD subjects except 22 who had increased flow. However, discrepant results were found by Zeng et al., who saw no difference in CBF, as assessed by arterial spin labelling, in 61 patients with BD [42].

*Microglia* Liu et al. found increases in 1 report and decreases in 2 [21].

Summary: the dominant changes in brain cell-type in BD are decreases in oligodendrocytes, neurons, and endothelial cells.

### Major depressive disorder (MDD)

*Astrocytes* In MDD, Liu et al. saw increases in 3 reports and decreases in 11 [21]; Oh et al. gave evidence for reduced numbers of astrocytes causing reduced uptake of glutamate from the synaptic cleft [43]; and Chandley et al. noted a robust decrease of astrocytic gene expressions [44]*.*

*Oligodendrocytes* In single reports, Liu et al. found both increases and decreases of oligodendrocytes [21]. In MDD, Aston et al. saw significantly decreased expression of 17 genes related to oligodendrocyte function [45]. Rajkowska et al. noted decreased oligodendrocyte somal size in the white matter of the ventral PFC [46].

*Neurons* Several reports showed decrease*s* in neuronal numbers [22, 35, 43, 47, 48]. One study showed a difference according to the presence or absence of early life adversity (ELA); MDD subjects who had suicided and also had exposure to ELA, had increased numbers of granule neurons in the dentate gyrus (DG) whereas without such ELA exposure there were fewer neural progenitor cells in the DG [49].

*Endothelial cells* Several reports show decreased cerebral blood flow in MDD [50–54].

*Microglia* Liu et al. saw increases in 5 reports and decreases in 1 [21].

Summary: the dominant changes in brain cell-type in MDD are decreases in astrocytes, oligodendrocytes, neurons, and endothelial cells, and increases in microglia.

### Schizophrenia

*Astrocytes* may be increased in SCZ [55, 56] but are sometimes decreased [57]*.*

*Oligodendrocytes* may be decreased in SCZ [58, 59]; a systematic study showed reduced levels in 15 of 25 reports [21].

*Neurons* are usually decreased in SCZ [60], with GABAergic neurons in the PFC fewer by 40% [61] (OK), non-pyramidal cells reduced by 40% in the hippocampal CA2 [62] (OK), and hippocampal neurons with reduced somal size [63].

*Endothelial cells* are often decreased in SCZ [64].

*Microglia* are usually increased in SCZ [65].

Summary: the dominant changes in brain cell-type in SCZ are decreases in oligodendrocytes, neurons and endothelial cells, and increases in microglia.

### AD

*Astrocytes in AD.* Emphasizing their importance for cognition, astrocyte numbers in the dentate gyrus were more reduced in Braak stages 3–4 than in stages 0–2 [66]; and because astrocyte processes wrap around cerebral microvasculature, the morphological modifications of astrocytes affect micro-cerebral blood flow [67] and, therefore, neural function [68].

*Oligodendrocytes in AD.* Mature oligodendrocytes perform myelination of naked axons; if their numbers are decreased, e.g., from impaired maturation of oligodendrocyte precursor cells (OPC), so that myelination becomes inadequate, then neural tracts suffer and cognition may be disturbed [68]. Exposure of oligodendrocyte cultures to 1 μM of Aβ_1-42_ induced cell death; morphological changes, e.g., shrunken cell bodies and a breakdown of their processes; and an 3.2-fold increase of lactate dehydrogenase activity released to the culture media [69].

*Neurons in AD* AD patients with severe tau pathology had a decreased number of newly generated neurons in their dentate gyrus [66]. Leng et al. found a selectively vulnerable subpopulation of excitatory neurons in the entorhinal cortex (EC) and showed a depletion of this subpopulation during AD progression [70].

*Endothelial cells in AD* Microvascular disease affects the progression of cognitive deficits in AD [71]. Endothelial cells contain the scavenge receptor, CD36, whose interaction with Aβ activates NADPH oxidase, producing vascular oxidative stress and neurovascular dysregulation [72]. String vessels, the remnants of destroyed microcapillaries, are seen throughout the brain of AD [18]. In addition, microinfarcts**,** with minute foci of neuronal loss as small as 50 µm, were seen in 43% of AD brains [73].

*Microglia in AD* Several reports concerning the role of microglia in AD are conflicting, the likely reason being the need to distinguish between proinflammatory and anti-inflammatory microglia, because a skewed M1 activation over M2 has been related to disease progression in AD [20], and this is the dominant but not universal change in AD.

Summary: the dominant changes in brain cell-type in AD are decreases in astrocytes, oligodendrocytes, neurons and endothelial cells, and increases in microglia.

### PTSD

*Astrocytes:* Gill et al. assessed astrocyte levels in PTSD and found them decreased [74]. Chronic stress in rats reduced the length and volume of astrocytic processes by 40.6% and 56%, respectively, and the number of their branch points was reduced by 57.8% [75].

*Oligodendrocytes:* Brains from females with PTSD had down-regulated oligodendrocyte-associated genes (*MBP* and *MBOP*) [76]. Bonnefil et al. showed that social defeat stress in mice reduced mature oligodendrocytes by 31.8% [77]. In another study they showed that chronic variable stress produced down-regulation of oligodendrocyte specific genes in the nucleus accumbens (NAcc) and PFC but upregulation in the corpus callosum [78]. Banasr et al. caused unpredictable stress in rats; after 15 days, the prelimbic cortex had 21% fewer oligodendrocytes [16]. After traumatic brain injury in rats, numbers of oligodendrocytes decreased significantly [79].

*Neurons:* Smith et al. produced fear conditioning in mice, causing reduced density of dendritic spines of pyramidal neurons in the hippocampus and medial PFC [80].

*Endothelial cells:* Brains from persons with PTSD had up-regulated endothelin1 gene, *EDN1*, cells in the vmPFC, which would decrease cerebral microvascular flow [76]. After traumatic brain injury in mice, there was a 1.5-fold increased apoptosis of cerebrovascular endothelial cells and increased BBB permeability [81].

*Microglia:* Fear conditioning also increased the length of microglial processes [80]. Rats whose behavior was severely disturbed by the scent of predators, had increased hippocampal microglia [82]. After single, prolonged stress, even minimal pain induced activation of hippocampal microglia [83].

Summary: the dominant changes in brain cell-type in PTSD are decreases in astrocytes, oligodendrocytes, neurons, and endothelial cells, and increases in microglia.

## Eleven available drugs benefit some or all of the five cell-types. Descriptions of their actions are deliberately brief: interested readers may consult the source citations for further information. N.B., use of any one of the following drugs for the purposes suggested in this article, would be off-label use

Highly abbreviated descriptions for likely therapeutic effects are provided here, showing either an increase that the drugs induce in number or activation of astrocytes, oligodendrocytes, synapses and neurons, and endothelial cells; or a decrease in number or activation of microglia.

**Clemastine** decreased loss of astrocytes [84]; increased postsynaptic proteins [84], muscarinic receptor, synapsin 1 and Homer 1, and improved oligodendrocyte survival/function [85, 86]; it enhanced myelin repair [87] and neuronal function [88]; it enhanced myelination in the PFC [14, 89]; it also enhanced visual function [86, 90]. Clemastine provided mitochondrial protection [91, 92]; and suppressed microglial M1 activation [93].

**Dantrolene,** by antagonizing ryanodine receptors and blocking Ca^2+^ release, prevents glutamate-caused increase in astrocytic volume [94], oligodendrocyte death [92], and preserves synaptic and neural function. Several other mechanisms for neuroprotection by dantrolene are in refs [95–106]. A potential adverse effect is on cerebral microvasculature, because release of BDNF requires Ca^2+^ mobilization [107].

**Erythropoietin** (EPO) increased differentiation of neuronal stem cells (NSC) into astrocytes [108]; increased oligodendrocytes [108], and neurons and dendritic spines [109] via increased production of brain-derived neurotrophic factor (BDNF) and its receptor [110]; and by enhancing differentiation of NSC [111]. EPO improved function of synapses,[112–114], endothelial cells [115, 116] and microglia [117].

**Fingolimod** an agonist of sphingomyelin phosphate (S1P), produces cell proliferation [118]. In astrocytes, fingolimod activated neurotrophic genes [119], and reduced formation of ceramide that causes apoptosis [120, 121]. It activated myelinating oligodendrocytes [122] increased OPCs, myelination, and neurological function [123], and ameliorated brain demyelination [124]. It enriched synaptic genes [125], prevented synaptic toxicity [126], and reversed synaptic hypersensitivities (123). It produced myelin in demyelinated brain [127], and prevented neural death from NMDA [128]. In multiple sclerosis, it improved axonal and myelin integrity [129]; and in mice prevented demyelination [130]. It increased dendritic spines [131, 132], reduced neuronal death from ROS [133], and improved mitochondrial production of ATP [134]. In microglia, fingolimod shifted M1 polarization toward M2 [135, 136].

**Fluoxetine** induced increases in: astrocytes [137], oligodendrocytes [138–140], neurons [138–140], endothelial cells [141], and decreased microglial activation [140, 142, 143].

Fluoxetine activates astrocytes to produce BDNF [137, 144, 145], and promotes clearance of astrocytes with damaged mitochondria [146]. It up-regulated OPC and oligodendrocyte markers [138], and reduced oligodendrocyte senescence [147]. Fluoxetine increased neurogenesis [138, 148], neuronal circuits [149], and spatial learning [150]. Neurons deprived of glucose and oxygen, had increased survival with fluoxetine [151]. It increased vasodilatation via differentiation and proliferation of endothelial cells and decreased arteriolar tone [141, 152]. In microglia, it attenuated NADPH oxidase activation, production of ROS and reactive nitrogen species, and down-regulated M1 and up-regulated M2 activation [5, 142, 153].

**Lithium** doubled astrocytic numbers and their VEGF secretion [154]. Oligodendrocyte expression of PLP and MBP increased, improving synaptic and neuronal function [155–159]. By negating activation of GSK-3β, synaptic expression of PSD-95 and gephyrin were enhanced. Lithium promoted neurogenesis [159–162]; doubled BDNF levels, increased dendritic length, increased anti-apoptotic Bcl2 and Bcl-_XL,_ and prevented neuronal death from glutamate and pro-apoptotic BAD, BAX, and caspase 3 [163]. It increased numbers and size of neural mitochondria [164], increased antioxidants [157, 164], minimized neurotoxicity from cytochrome c, and promoted mitochondrial biogenesis [165, 166]. Lithium benefitted microvascularity by increasing VEGF secretion [167] and BBB integrity [168]. Lithium’s inhibition of GSK-3β, reduced production and activation of pro-inflammatory mediators [169–171].

**Memantine** induced activation of astrocytes [172], and prevented losses of oligodendrocytes [173], synapses and neurons [174–176], and endothelial cells [177]. It is a NMDAR antagonist, enters the NMDAR’s Ca^2+^ channel and decreases its permeability, so prevents neuronal excitotoxicity and death [174]. Memantine provides synaptic protection via several mechanisms (183,184,185,186,187): preventing cytotoxicity by blocking inhibition of a guanisine triphosphatase involved in multiple cellular processes, thus protecing against mitochondrial dysfunction causing cell death via cytochrome c release, ROS and peroxide production [178–181]. Blocking the ion channel of the acetylcholine receptors also prevents neurotoxicity [182]. Memantine benefitted brain endothelial cells and blocked disruption of the BBB [183].

**Minocycline** prevented oligodendrocyte toxicity caused by deprivation of oxygen and glucose [184], microglial-induced apoptosis [185], by minocycline preventing inhibition of CREB’s up-regulation [186] and caused by Aβ [187]. Synaptic function improved from increased levels of PSD-95 and dendritic spines [188]. It prevented cognitive decline caused by an antagonist of the NMDAR [189]. Neuronal benefit was also from its increased expression of BDNF, CREB, and phospho-CREB [186], and proliferation of NPCs [190]. Minocycline potentiated neurite outgrowth [191]. In a transgenic mouse model of Down’s syndrome, minocycline prevented decline of cholinergic neurons [192]. In a list of 1040 drugs that prevent release of cytochrome c, minocycline was the second most potent [193], the mechanism involves inhibiting mitochondrial increases in Ca^2+^ concentration plus inhibition of NADH-cytochrome c reductase and cytochrome c oxidase [193, 194]. Finally, minocycline prevented microglial activation [195, 196].

**Pioglitazone**, a PPARγ agonist, prevents phosphorylation of JAK-STAT in astrocytes and thereby induces increases in neurons [197, 198], oligodendrocytes [153, 198], endothelial cells [199], and decreases in microglia [200]. Ciglitazone and curcumin, also PPARγ agonists, were cytoprotective for astrocytes and reversed their decreased expression of PPARγ receptor after exposure to Aβ_25-35_ [201, 202]; and ciglitazone increased formation of oligodendrocyte progenitors [198]. Pioglitazone rescued the demyelination caused by anti-MOG autoantibody [203]. The promotion of OPC differentiation into mature oligodendrocytes by IL-4 was mediated by PPARγ or curcumin [204, 205]. PPARγ agonism increases the neurogenic differentiation gene, *NeyroD1* [198], and protected cortical neurons and axons against toxicity induced by NO or KCl-induced toxicity [197, 206].

PPARγ also produces endothelial cell proliferation, and angiogenesis [199, 207]. In microglia, PPARγ agonists inhibit cytokine production by down-regulating proinflammatory genes(211). In addition, rosiglitazone up-regulated M2 microglia [208].

**Piracetam** increased numbers and function of astrocytes [209]. It also increased function of synapses [210–213] and neurons [210]: it decreased neurotoxicity from deprivation of oxygen and glucose, hypoperfusion, ethanol feeding or ethanol withdrawal [211, 214–216] It also caused longer neurites [210]. The effects of piracetam may derive in part from the restoration of cell membrane fluidity induced by a conformational change in the phospholipids of liposomal membrane [213]

By decreasing mitochondrial swelling and permeability caused by excessive Ca^2+^opening the MPTP, it improved mitochondrial membrane potential and ATP levels, shifted the balance of mitochondrial fission or fusion towards fusion, and reversed the adverse effect of pro-oxidants [217]. Additionally, it reversed the cytotoxic effects of p53 and BAX [211].

**Riluzole** increased both the gene for the excitatory amino acid transporter (EAAT2) and its expression, thus enhancing glutamate uptake by astrocytes, and protecting against excitotoxicity of neurons [218, 219]. It benefits synapses, by inhibiting voltage-activated sodium currents which prevents reverse operation of the Na^+^/Ca^2+^ exchanger [220], and by increasing LTP [221]. It caused a 40-fold increase in hippocampal BDNF, creating neurogenesis [222]; and protected against neural degeneration caused by ischemia [223]. N-acetylaspartate is exclusively expressed in neurons and was increased in the cerebral cortex by exposure to riluzole [224]. In neurons, it also decreased oxidative stress, lipid peroxidation, and ATP depletion [225, 226]. Microglial activation was ameliorated by riluzole [227], which also upregulated the mRNA levels of M2 markers and downregulated those of M1 markers [228].

## Drugs that might be therapeutically beneficial in a reasonable number of patients, by addressing the dominantly affected cell-types in BD, MDD, SCZ, AD, and PTSD

*For BD*, increasing oligodendrocytes, neurons and endothelial cells (CBF), is achieved by erythropoietin, fluoxetine, lithium, memantine, and pioglitazone.

*For MDD,* increasing astrocytes, oligodendrocytes, neurons, and endothelial cells (CBF), and decreasing microglia, is achieved by erythropoietin, fluoxetine, lithium and pioglitazone.

*For SCZ*, increasing oligodendrocytes, neurons, and endothelial cells (CBF), and decreasing microglia, is achieved by erythropoietin, fingolimod, fluoxetine, lithium, and pioglitazone.

*For AD,* increasing astrocytes, oligodendrocytes, neurons, and endothelial cells (or CBF), and decreasing microglia, is achieved by erythropoietin, fluoxetine, lithium and pioglitazone.

*For PTSD,* increasing astrocytes, oligodendrocytes, neurons, and endothelial cells, and decreasing microglia, is achieved by erythropoietin, fluoxetine, lithium and pioglitazone.

### Shared dominant cell-types between disorders

There are shared changes in dominant cell-types between some psychiatric conditions, from which one may infer shared pathogenesis and similar treatments. Thus, MDD, AD, and PTSD, share decreases in astrocytes, oligodendrocytes, neurons, and endothelial cells, and increases in microglia. SCZ has decreases in oligodendrocytes, neurons, and endothelial cells, and increases in microglia, so shares these features with MDD, AD, and PTSD. Evidence of an overlapping genetic risk that is similar to their overlapping of affected cell-types, was shown for MDD and AD by Lutz et al. [229], between PTSD and schizophrenia by Duncan et al. [230], and between MDD and schizophrenia by The Psychiatric Genomics Consortium [231]. That consortium demonstrated significant genetic correlations between SCZ and BD (0.68), MDD and BD (0.47), SCZ and MDD (0.43), and was non-significant between psychiatric disorders and the negative control, Crohn’s disease. The foregoing genetic data support the concept proposed in this article, that brain cell-types, may be used as surrogates for all of the genetic, metabolic, and environmental, data for the inputs of which they are storehouses.

### APOE4 affects functions of astrocytes, oligodendrocytes, neurons, endothelial cells, and microglia

Further support for the concept that brain cell-types may be used as surrogates for several abnormalities affecting their function, comes from brain cell-types that carry APOE4. The APOE4 variant of the *APOE* gene product, renders its carriers susceptible to AD. One mechanism for this may be its effect on the five families of brain cell-types. Blanchard et al. reported several such effects: that cholesterol transport was defective in oligodendrocytes derived from induced pluripotent stem cells (iPSC) carrying APOE4; that these APOE4+oligodendrocytes accumulated cholesteryl ester species; and that this was associated with down-regulation of genes linked to myelination and decreased production of MBP [232]. Lin et al. showed that the APOE4 variant can also lead to extensive gene expression alterations in neurons, astrocytes, and microglia; APOE4 astrocytes had cholesterol accumulation, and APOE4 microglia-like cells had increased up-regulation of genes associated with immune responses and inflammation [233]. Lipid accumulation in APOE4+microglia was seen also by Victor et al.; and, as compared with that of APOE3 microglia, the pro-inflammatory activity of APOE4+microglia was exacerbated by medium from neuronal cultures, demonstrating cross-talk between neurons and APOE4+microglia [234]. APOE4+endothelial cells had dysfunctions in pathways leading to blood clotting factors and inflammation [235]. Finally, removal of APOE4 from astrocytes led to improvements in synaptic function with increase in PD95, neurons, oligodendrocytes, and microglia [236]. Thus, all the foregoing data suggest that one of the several mechanisms accounting for the association between APOE4 and AD, is that APOE4 detrimentally affects the functions of astrocytes, oligodendrocytes, synapses, neurons, endothelial cells, and microglia, which supports the thesis of this article, that treatments should be directed to improving the functions of affected brain cell-types.

### Adverse effects

The choice of a medication is partly determined by Its safety profile, particularly for serious adverse events (SAEs), less so for minor adverse events (AE). Following are summaries of reports of SAEs for the above drugs.

Clemastine: no SAEs seen in several clinical trials.

Dantrolene: other than caused by preexisting disease, SAEs were rare [237].

Erythropoietin: only 3 of 50 studies attributed SAEs to erythropoietin but those used very high-dosages (> 200,000 IU) [238]. If chosen for use in patients with BD, MDD, or SCZ, erythropoietin should be low dosage, 10,00 IU given IM once monthly.

Fingolimod: pooled data from 1640 subjects, showed that fingolimod 0.5 mg gave transient, usually asymptomatic, second-degree heart block, on treatment initiation; minor BP increases; generally asymptomatic liver enzyme elevations (9%); and macular oedema (0.4%) [239]. Higher doses (1.25 mg) gave more frequent SAEs. In patients with BD, MDD, SCZ, AD, dosage should be only 0.5 mgs daily.

Fluoxetine: Beasely et al. obtained data from 25 double-blind clinical trials involving 4016 patients with MDD randomized to treatment that included fluoxetine 20 to 80 mg/d [240]. At a dose of 20 mg/d, fluoxetine-treated patients had a discontinuation rate due to adverse events that was not statistically significantly different from that in placebo recipients. Nevertheless, although in this article the suggested dose is only 10 mg/d in combination with lithium, caution is necessary because the possibility exists of inducing mania, even though that is reported mainly with doses of ≥ 20 mg/day [241].

Lithium: SAEs, include hypercalcemia, hypothyroidism [242], nephrogenic diabetes insipidus, and renal insufficiency [243]. Low dosage (< 100 mgs daily) benefited Alzheimer’s dementia; thus 75 mg daily might be an appropriate dose in BD, MDD, SCZ, AD or PTSD. It should not be used if glomerular filtration rate is < 60 mL/min.

Memantine: no significantly increased SAEs [244].

Minocycline: a review showed six patients with a hypersensitivity syndrome, six with serum sickness, and 24 with drug-induced lupus [245]. Minocycline has been used in thousands of persons over manyyears, so SAEs are rare. ANA test should be negative before prescribing it.

Piracetam: a large study, did not show more SAEs than with placebo [246].

Riluzole: nine studies did not show increased SAEs [247].

### The need for clinical trials

Clinical trials would demonstrate validity of both the proposed concept and the choice of drugs for treatment; and would also show the percentage of patients benefitting from the chosen combination of drugs administered for a particular psychiatric condition. In a clinical trial that randomly assigns participants to receive active drugs or placebo, the primary objective would be to show ≥ 30% better results from active drugs than from placebo. In order to provide deeper coverage and lower dosages, two drugs rather than one should be used.

## Discussion

The proposed model is a basis for formulating treatment of major neuro- psychiatric conditions. The model overcomes the problem of addressing the many genetic, mitochondrial, and metabolic impairments associated with the discussed conditions; it proposes drugs that address the dominant combination of brain cell-types, which simplifies use of known data; future availability of data that involve subtypes of brain cell-types, will allow improvement of the model. Drugs are discussed which address the brain’s five cell-types that affect the occurrence of BD, MDD, SCZ, AD, and PTSD.

It is acknowledged that a single treatment combination would not benefit all patients with a particular psychiatric phenotype, because an identical clinical phenotype may result from several, different combinations of cell-types, only one of which is dominant compared with the other as compared with the others; thus, the suggested treatments would benefit only those subjects whose condition is caused by a particular set of dominant cell-types. Nevertheless, benefit to even 20–30% of patients would be clinically useful. It is, also, worth noting that in studies of all of the mentioned conditions, ‘conflicting’ results are reported; yet because an identical clinical phenotype may incorporate different combinations of cell-types, it is possible that the different results might not be actually ‘conflicting’ but correct.

The determination of the dominant change in the brain cell-type in any particular condition is based upon the findings in published reports and, thus, the diligence with which the literature is reviewed. Data for the present article were derived primarily from citations provided by PubMed and Google Scholar. Citations found in key articles are often very relevant; for example, ref [60] cited an article that reviewed studies in 1415 patients with SCZ [248]. For the four psychiatric conditions used to illustrate the proposed approach, the 61 citations included seven that reviewed 1136 separate, published reports [21, 23, 37, 41, 56, 60, 68], and one that reviewed 15 publicly available data sets [37]. Reviews generally refer to very large numbers of subjects; e.g., one “review of reviews” analyzed 683 reports in 26 reviews involving 55,561 patients [65].

It is relevant to ask, ‘which is primary, alterations in brain cell-types or the changed behavior?’ because if it is the altered behavior that causes the changes in brain cell-types, then addressing the changes in those cells would not affect behavior. However, and fortunately for the thesis of this article, a number of studies show that the altered brain cells precede the altered behavior. In one such study, Santarelli et al. noted that chronic antidepressant treatments increase adult hippocampal neurogenesis; they found that destruction by X-irradiation of the region of mouse brain containing the hippocampus, prevented both the neurogenesis and the behavioral benefits of two classes of antidepressants, showing that stimulation of neurogenesis in the hippocampus underpins the behavioral effects of chronic antidepressants [249]. Related to that, the induction of depression-like behavior in chronic stress models was associated with elimination of postsynaptic dendritic spines and a loss of PFC projection neurons [250]; and ablation of astrocytes in the PFC, which deprives neurons of trophic support, increased anhedonia, anxiety, and helplessness, similar to the depression-like behaviors caused by chronic unpredictable stress [251]. The above studies may be interpreted as demonstrating that. for the relationship between neurons and behavior, it is the neuronal action that precedes a behavioral change, not vice versa.

There are, of course, many caveats to consider: (1) Cell-type families have sub-types; therefore, designating a drug as addressing a generic cell-type may be inaccurate. However, there are scores of subtypes. E.g., Tasic et al. found 56 glutamatergic and 61 GABAergic, cortical neuronal subtypes [252], and Bickoff et al. documented multiple subtypes of spinal, inhibitory interneurons [253]. Currently, data are sparse concerning the presence and distribution of cell-subtypes in the major psychiatric conditions, and there is no information regarding a possible, differential effect of medication upon subtypes. In future, when more information about them becomes available, it will be possible to establish if subtypes must be separately accounted for. (2) One report found no consistent evidence of astrocytic impairments in 11 psychiatric disorders; that study determined cell-type by using single cell transcriptomic data integrated with genome wide association study results [254]. However, there is variability of gene expression by astrocytes, e.g., Stahlberg et al. identified two subpopulations of astrocytes with distinct gene expression profiles [255]. Thus, absence of either a specific gene or set of genes would incorrectly label some cells as being ‘not astrocytes’. Further, glial fibrillary acidic protein (GFAP) is the cardinal cytological marker of astrocytes but GFAP-negative astrocytes also exist, e.g., Tatsumi et al. saw that brain regions such as the basal forebrain, thalamic nuclei, and deep cerebellar nuclei, tended to lack GFAP-positive astrocytes [256]; thus also in this case, the absence of GFAP would incorrectly identify cells as being ‘not astrocytes’. (3) Another limitation of the proposed algorithm is that it assumes uniform changes of cell-types across all brain regions. That this is not necessarily the case, is exemplified by observations made by Gandal et al. in brains from patients with autistic spectrum disorder (ASD) [257]. They saw a regional gradients, with three-fold to four-fold more differentially expressed genes in cell-types (particularly excitatory neurons, interneurons, and oligodendrocytes) in occipital and parietal cortices than in the PFC.

Despite these theoretical limitations there is, nevertheless, theoretical consistency in that the proposed target of therapy is the changed direction of number or function of the individual cell-types as seen in individual psychiatric conditions. The accuracy of the cell-type(s) as target can be tested by clinical trial since knowing the changed direction of cell-types provides clinical benefit if a specific drug or combination of drugs corrects both the changed direction and the clinical condition.

## Conclusion

1. For most psychiatric conditions, pathogenesis depends on impairment of either number or function among the five families of cell-types in the brain. Cell-types are storehouses of and, therefore, surrogates for, pathogenically important genetic, metabolic, and environmental inputs. The dominantly changed cell-types in a given condition permits algorithmic formulation of a pharmacotherapeutic regimen.
2. Because an identical clinical phenotype may result from different combinations and permutations of impaired cell-types, no single combination of cell-types accounts for all cases; in a given set of subjects one can only discern the combination that is dominant as compared with the others; therefore, different studies may produce discrepant results, all of which could be accurate.
3. Eleven drugs are presented that benefit either all or some of the five cell-types and from among which treatment may be formulated for each condition.
4. Clinical trial is required to validate both the proposed concept and choice of drugs.

## Data Availability

Data regarding the suggested drugs are available from the author.
